# Respiratory-swallow coordination training using bimodal signal biofeedback for patients with post-stroke dysphagia: a randomized controlled trial

**DOI:** 10.1080/07853890.2025.2607218

**Published:** 2025-12-29

**Authors:** Lian Wang, Jia Qiao, Zhenhai Wei, Xiaoqin Liu, Xiaomei Wei, Zulin Dou

**Affiliations:** a Department of Rehabilitation Medicine, Union Hospital, Tongji Medical College, Huazhong University of Science and Technology, Wuhan, Hubei, China; b Department of Rehabilitation Medicine, The Third Affiliated Hospital of Sun Yat-sen University, Guangzhou, Guangdong, China; c Department of Rehabilitation Medicine, The Third Affiliated Hospital of Southern Medical University, Guangzhou, Guangdong, China; d Department of Rehabilitation Medicine, West China Second University hospital, Sichuan University, Sichuan, China

**Keywords:** Stroke, dysphagia, respiratory-swallow coordination, biofeedback

## Abstract

**Objective:**

The purpose was to investigate the effects of respiratory-swallow coordination training with bimodal signal biofeedback on swallowing function in patients with post-stroke dysphagia.

**Methods:**

Post-stroke dysphagia Patients were randomly assigned to either the control group or the experimental group. The control group received conventional rehabilitation, while the experimental group underwent additional respiratory-swallow coordination training based on biofeedback. The training protocol consisted of three phases, conducted at an intensity of 30 min/day, 6 days/week, for two consecutive weeks. Outcome measures included the Functional Oral Intake Scale (FOIS) score, the Rosenbek Penetration-Aspiration Scale (PAS) score, respiratory-swallow coordination, and videofluoroscopic swallowing study temporal and kinematic parameter. Assessments were conducted at baseline, post-treatment, and at a one-month follow-up.

**Results:**

Thirty patients were enrolled. Both groups showed significant improvement in FOIS scores from baseline to both two-week post-treatment and one-month follow-up (*p* < 0.001). Compared to the controls, the experimental group demonstrated significantly greater FOIS scoreimprovement at both post-treatment and follow-up (*p* < 0.001). The proportion of patients with *a* ≥ 2-point increase in FOIS scores was significantly higher in the experimental group than in the control group at both post-treatment (*p* < 0.01) and one-month follow-up (*p* < 0.01). After two weeks of treatment, the percentage of PAS scores ≥6 was significantly lower in the experimental group than in the control group (*p* < 0.001). Additionally, the percentage of optimal respiratory-swallow pattern was significantly higher in the experimental group than in the control group (*p* < 0.001).

**Conclusion:**

Bimodal signal biofeedback-based respiratory-swallow coordination training can effectively improve respiratory-swallow coordination and swallowing function in patients with post-stroke dysphagia.

## Introduction

Swallowing and respiration are essential physiological activities for sustaining life. They share certain anatomical structures and are regulated by central pattern generators (CPGs) in the brainstem [1–3]. Swallowing and respiration are highly coordinated. The physiological basis of their coordination lies in the interaction between the swallowing CPG and the respiratory CPG [4]. This results in a brief, well-timed pause in respiration—known as swallowing apnea (SA)—to accommodate the swallow, which typically occurs during the expiratory phase and is often followed by a brief post-swallow expiration [5–9]. During swallowing, the respiratory-swallow pattern, defined by respiratory phases before and after SA, is a key indicator of respiratory-swallow coordination.

Previous research using various techniques has identified four types of respiratory-swallowing patterns: exhale-swallow-exhale (E-SW-E), exhale-swallow-inhale, inhale-swallow-exhale, and inhale-swallow-inhale [10]. Among these, E-SW-E is the most common, occurring in 71% to 100% of cases [11]. In this pattern, swallowing starts during expiration and is followed by a brief expiratory phase. Studies have shown that initiating swallowing during expiration helps generate subglottic positive pressure, thereby effectively reducing the risk of food and liquid entering the airway [12]. Moreover, a study using videofluoroscopic swallowing study (VFSS) and fiberoptic endoscopic evaluation of swallowing found that swallowing initiated during the expiratory phase facilitates critical physiological movements, including tongue base retraction, pharyngeal contraction, laryngeal elevation, laryngeal vestibule closure, and vocal fold adduction [13]. Additionally, it is easier to understand that exhaling after swallowing, rather than inhaling immediately, helps further reduce the risk of aspiration. Therefore, the E-SW-E pattern is considered the most favorable for ensuring swallowing safety and efficiency [14].

Patients with post-stroke dysphagia often have impaired respiratory-swallow coordination [15]. Compared with healthy controls, they demonstrate a markedly greater percentage of swallows that interrupt the inspiratory phase, leading to a higher prevalence of non-protective patterns, particularly the inhale-swallow-inhale pattern [16,17]. Given the importance of respiratory-swallow coordination in swallowing safety, timely and targeted interventions to restore the E-SW-E pattern are crucial for promoting swallowing function recovery and reducing the risk of aspiration [18]. However, patients often struggle to intuitively perceive their respiratory-swallow coordination during training, which may ultimately reduce the effectiveness of respiratory-swallow coordination therapy.

Recently, biofeedback technology has become increasingly used in swallowing rehabilitation [19–21]. It offers real-time monitoring and feedback of physiological signals, helping patients intuitively perceive their movements and make necessary adjustments to optimize motor output [22]. A growing body of evidence indicates that biofeedback-assisted swallowing therapy is effective in improving swallowing function [23–25]. The practical application of this strategy commonly involves non-invasive sensors, such as accelerometers and respiratory sensors, to capture swallowing and respiratory signals [26]. Some researchers have explored integrating biofeedback technology with respiratory-swallow coordination training for treating dysphagia. For example, Martin-Harris et al. [27] used respiratory sensors to provide real-time biofeedback during respiratory-swallow coordination training, helping patients in re-establishing the E-SW-E pattern. This intervention led to improvements in both respiratory-swallow coordination and swallowing function in individuals with head and neck cancer. However, the single-arm design without a control group limited the study’s ability to rigorously evaluate the effectiveness of respiratory-swallow coordination training. Additionally, the intervention’s efficacy may vary depending on the underlying disease, and treatments effective for dysphagia caused by one condition may not work for others [28]. Therefore, further research is warranted to investigate the therapeutic efficacy of biofeedback-based respiratory-swallow coordination training for post-stroke dysphagia.

Respiratory-swallow coordination training requires precise coordination between respiration and swallowing. To enhance the precision of this training, we developed a bimodal biofeedback system. This system extends the established respiratory biofeedback protocol by Martin-Harris et al. [27] by combining a nasal cannula with a triaxial accelerometer, which provides patients with synchronous visual feedback for both the swallowing event and the respiratory cycle. We hypothesize that this combined feedback offers a more integrated cue than respiratory feedback alone, enabling patients to better perceive the target exhale-swallow-exhale pattern. This study aimed to investigate the effects of bimodal biofeedback-based respiratory-swallow coordination training on respiratory-swallow coordination and swallowing function in patients with post-stroke dysphagia.

## Materials and methods

This prospective randomized controlled trial was conducted as part of a registered project in the Chinese Clinical Trial Registry (ChiCTR2300068908) at the Third Affiliated Hospital of Sun Yat-sen University from March 2023 to December 2024. The study was approved by the hospital’s Ethics Committee (no. [2022]02-192-01) and conducted in accordance with the Declaration of Helsinki. All participants provided written informed consent prior to participation.

### Participants

Thirty participants were enrolled in the study. The inclusion criteria were as follows: (1) diagnosis of first-time ischemic or hemorrhagic stroke confirmed by MRI or CT; (2) stroke duration of less than 6 months; (3) diagnosis of dysphagia confirmed by VFSS, defined by the presence of any one of the following: airway penetration or aspiration; (4) the percentage of E-SW-E pattern is less than 40%; (5) no tracheostomy; (6) age between 18 and 80 years, irrespective of gender. The exclusion criteria were as follows: (1) dysphagia caused by other conditions, such as nasopharyngeal cancer or Parkinson’s disease; (2) history of respiratory diseases affecting lung function, such as chronic obstructive pulmonary disease.

Participants were randomly assigned to either the control group or the experimental group. The control group received conventional training, while the experimental group received the same conventional training with the addition of biofeedback-based respiratory-swallow coordination training. All participants received conventional swallowing rehabilitation training for 45 min/day, 6 days/week, for 2 consecutive weeks. The training protocol included the following components: (1) oral sensory facilitation using cold tactile stimulation applied to the tongue and palate arch; (2) tongue strength and coordination exercises, including active tongue protrusion, lateralization, lip-licking, and hard palate pressing, combined with tongue resistance training; (3) swallowing maneuver training incorporating chin-tuck swallow, supraglottic swallow, Mendelsohn maneuver, effortful swallow, and head-turn swallow. The subjects were allowed a few minutes of rest between exercises. All training was conducted under the guidance of the speech-language therapists and was individually adjusted according to each patient’s tolerance and clinical condition. Both groups received conventional swallowing rehabilitation from the same therapist team.

### Biofeedback treatment

**Figure 1.**
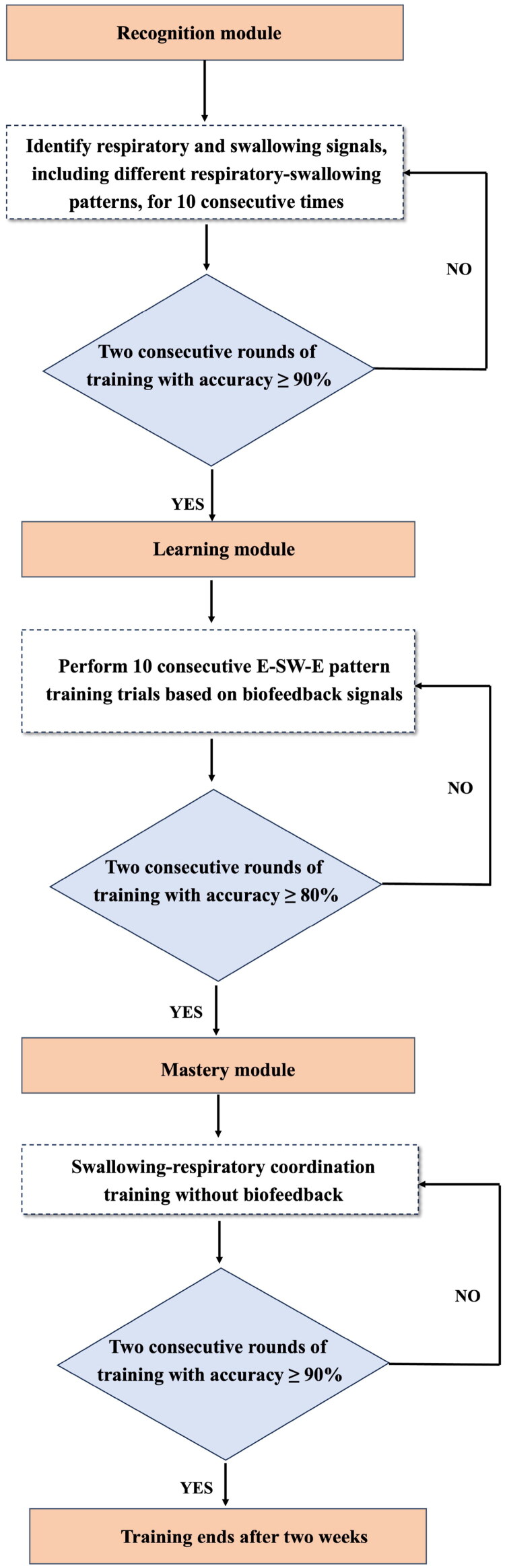
Flowchart of respiratory-swallow coordination training.

In this study, the respiratory-swallow coordination training protocol was adapted from the protocol developed by Martin-Harris et al. [27]. A key adaptation was the implementation of a bimodal biofeedback system. The biofeedback system integrates an accelerometer (ADXL357, Analog Devices) and a nasal airflow sensor (AFM3000-200, Guangzhou Aosong Electronics Co., Ltd., China) to enable synchronized monitoring of swallowing and respiration signals. The accelerometer was secured at the anterior cricoid cartilage level using medical adhesive tape [29], while the nasal cannula, connected to the nasal airflow sensor, was placed at the nostrils. The training regimen consisted of 30 min/day, 6 days/week, for 2 consecutive weeks. The training comprised three modules: recognition, learning, and mastery, detailed as follows (Figure 1):

Recognition module: Participants were first shown pre-recorded biofeedback signals illustrating exhalation, inhalation, and SA during quiet breathing. Specifically, an upward curve in the respiratory signal indicated exhalation, a downward curve represents inhalation and a flat line during swallowing indicates SA. They were then introduced to the E-SW-E pattern before observing real-time respiratory and swallowing signals to identify these features. Advancement to the next module required at least 90% accuracy in two consecutive rounds, correctly identifying features in at least 9 out of 10 trials.

**Figure 2.**
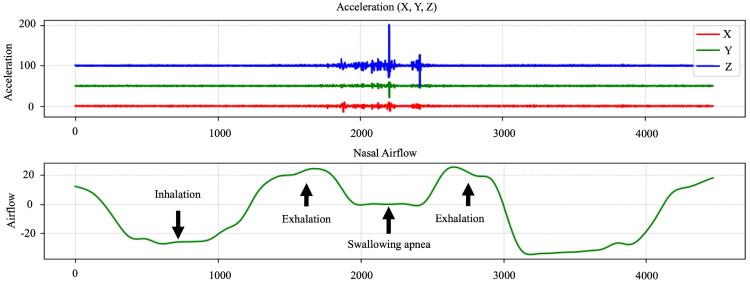
Representative signals of the Exhale–Swallow–Exhale (E-SW-E) pattern.

Learning module: Participants wore a nasal cannula and an accelerometer to observe real-time respiratory and swallowing signals while training with biofeedback. They were instructed to initiate swallowing during mid-to-late exhalation and finish with a brief exhalation (Figure 2). During training, therapists provided verbal prompts as needed. To advance, participants had to complete at least 8 out of 10 swallowing tasks correctly without prompts and maintain ≥80% accuracy for two consecutive rounds.

Mastery module: Participants continued wearing sensors but no longer received real-time biofeedback. They practiced independently based on the learned E-SW-E pattern, while therapists monitored their respiratory and swallowing signals for accuracy. Successful completion of this stage required participants to demonstrate at least 90% accuracy across two consecutive training sessions.

Whether therapeutic feeding training was included during the learning and mastery modules depended on the participants’ VFSS assessment results. If the Penetration-Aspiration Scale (PAS) score for swallowing a specific bolus consistency was ≤ 6, the participant could perform oral feeding training with that consistency during the training; if the PAS score exceeded 6 for all consistencies, a moistened cotton swab was used to minimize the impact of dry mouth on swallowing initiation. Daily training reinforced previously completed modules, with a primary focus on the mastery module once the recognition and learning criteria were met.

### Outcome assessment

The primary outcome was the Functional Oral Intake Scale (FOIS) score, which ranges from level 1 (nothing by mouth) to level 7 (total oral intake with no restrictions) [30]. Secondary outcomes included the PAS score, which ranges from level 1 (material does not enter the airway) to level 8 (material enters the airway, passes below the vocal folds, and no effort is made to eject it) [31]. For each patient, we calculated the proportion of swallows with PAS scores ≥ 6 across all swallows completed during the VFSS examination. The VFSS protocol comprised four barium sulfate suspensions adhering to International Dysphagia Diet Standardization Initiative (IDDSI) levels 0 (thin) to 3 (high-viscosity), administered in a fixed sequence: medium viscosity (3, 5, 10 ml), followed by low viscosity (3, 5, 10 ml), then thin liquid (3, 5, 10 ml), and finally high viscosity (3, 5, 10 ml). The decision to proceed was made based on the patient’s condition. Additional secondary outcomes included respiratory-swallow coordination assessment and VFSS temporal and kinematic parameters, measured during the swallowing of 5 mL of moderately thick liquid. The measured temporal parameters included: (1) velar elevation duration, defined as the interval from the forward movement of the soft palate from its starting position to the end of its backward and downward motion; (2) laryngeal vestibule closure duration, defined as the period from the first to the last contact between the inferior surface of the epiglottis and the arytenoids; and (3) upper esophageal sphincter (UES) opening duration, measured from the onset of UES opening until the bolus tail passed through the UES and the UES began to close. The kinematic parameters comprised: (1) pharyngeal constriction ratio, a validated surrogate measure of pharyngeal strength calculated as the ratio of the pharyngeal area at maximal constriction to the pharyngeal area in the hold position; and (2) UES opening width, measured at the maximum distention during the swallow. These parameters were defined and calculated following established protocols for VFSS analysis [32,33].

PAS scores, VFSS parameters, and respiratory-swallow coordination were assessed at baseline (T0) and immediately after the two-week intervention (T1). The FOIS scores were assessed at T0, T1, and one month following the intervention (T2). All outcome assessments were conducted by well-trained raters who were blinded to both participant group allocation and assessment time points.

### Statistical analysis

The Shapiro-Wilk test was used to assess the normality of the data. Normally distributed data were presented as mean ± standard deviation (mean ± SD), while non-normally distributed data were expressed as the median and interquartile range (M (Q1, Q3)). Between-group comparisons: Independent samples t-test was used for normally distributed data, whereas the Mann-Whitney U test was applied for non-normally distributed data. Within-group comparisons: Paired t-test was used for normally distributed data, while the Wilcoxon signed-rank test was applied for non-normally distributed data. For variables measured multiple times within a group, repeated measures ANOVA was used if homogeneity of variance was met; otherwise, the Friedman test was performed. Categorical data were presented as frequency and percentage and analyzed using the chi-square test or Fisher’s exact test. *p* < 0.05 was considered statistically significant. For between-group comparisons of FOIS scores at post-intervention (T1) and follow-up (T2), the Mann-Whitney U test was performed separately for each time point. Considering multiple testing correction, *p* < 0.025 was considered statistically significant. IBM SPSS Statistics (version 26) and GraphPad Prism (version 8) software were used for data analysis and graphing.

## Results

A total of 30 patients were enrolled in the study. The control group comprised 15 patients (13 males; mean age: 60.93 ± 12.69 years), and the experimental group also consisted of 15 patients (13 males; mean age: 59.67 ± 11.67 years) (Table 1).

**Table 1.** Baseline characteristics of the two groups.

Variables	Control group (*n* = 15)	Experimental group (*n* = 15)	t	df	*P-*value
Age (year), mean ± SD	60.93 ± 12.69	59.67 ± 11.67	0.285	28	0.778
Sex			–	–	1.000
male, n (%)	13 (87%)	13 (87%)			
Time from onset (month), M (Q1, Q3)	2 (1, 4)	3 (2, 4)	–	–	0.239
stroke type			–	–	0.651
infarction, n (%)	11 (73%)	13 (87%)			
hemorrhage, n (%)	4 (27%)	2 (13%)			
ADL, mean ± SD	41.33 ± 16.53	52.67 ± 19.88	−1.692	28	0.102
mRS, M (Q, Q3)	4 (3, 4)	3 (3, 4)	–	–	0.143
Comorbidity					
hypertension, n (%)	10 (67%)	14 (87%)	–	–	0.169
diabetes mellitus, n (%)	6 (40%)	6 (40%)	–	–	1.000
CHD, n (%)	2 (13%)	2 (13%)	–	–	1.000
FOIS score, M (Q1, Q3)	1 (1, 1)	1 (1, 1)	–	–	0.654

ADL, Activities of Daily Living; mRS, Modified Rankin Scale; CHD, Coronary Heart Disease; FOIS, Functional Oral Intake Scale.

In the control group, FOIS scores differed significantly across the pre-treatment, two-week post-treatment, and follow-up assessments (*p* < 0.001). Bonferroni post hoc analysis indicated that FOIS scores significantly improved at two weeks post-treatment compared to baseline (adjusted *p* = 0.004) and showed a significant improvement at follow-up compared to baseline (adjusted *p* < 0.001); however, the difference between follow-up and two weeks post-treatment was not statistically significant (adjusted *p* = 0.604).

**Figure 3.**
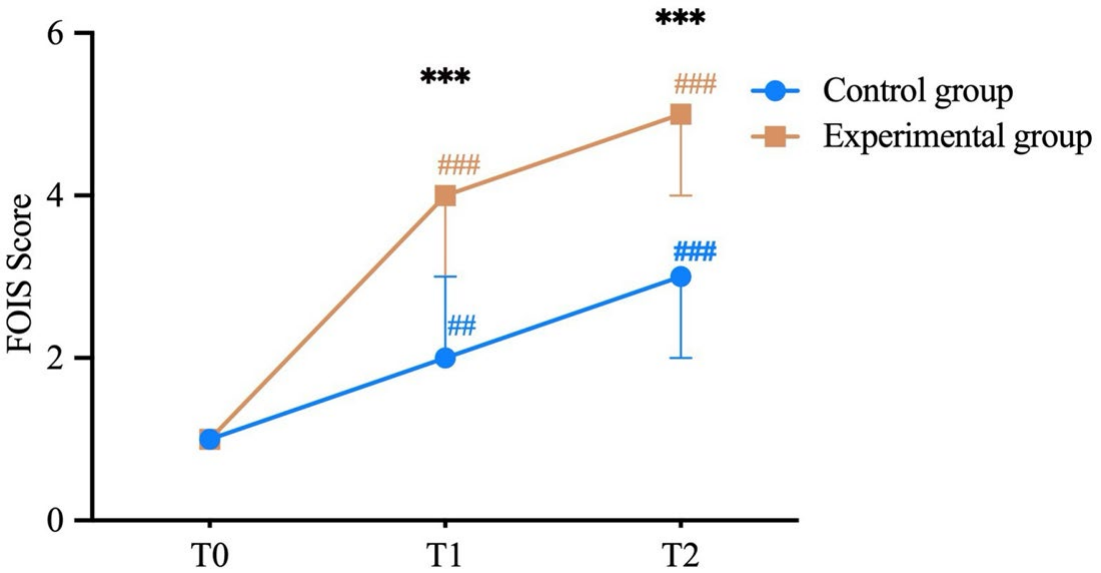
FOIS scores of the two groups at different assessment time points. T0 indicates baseline; T1 indicates after two weeks of intervention; T2 indicates follow-up. Orange ‘#’ symbols denote within-group comparisons (vs. T0) for the experimental group, while blue ‘#’ symbols denote within-group comparisons (vs. T0) for the control group. ‘##’ indicates *p* < 0.01 and ‘###’ indicates *p* < 0.001. Black ‘*’ symbols represent between-group comparisons at each time point (T1 and T2), with ‘***’ indicating *p* < 0.001. For both groups, the differences in FOIS scores between T2 and T1 were not statistically significant.

In the experimental group, FOIS scores differed significantly across the pre-treatment, two-week post-treatment, and follow-up assessments (*p* < 0.001). Bonferroni post hoc analysis showed that FOIS scores significantly improved at two weeks post-treatment compared to baseline (adjusted *p* = 0.010) and were significantly higher at follow-up compared to baseline (adjusted *p* < 0.001); although FOIS scores at follow-up were higher than at two weeks post-treatment, the difference was not statistically significant (adjusted *p* = 0.053). Additionally, FOIS scores in the experimental group were significantly higher than those in the control group at both two weeks post-treatment and follow-up (*p* < 0.001) (Figure 3). The proportion of patients with *a* ≥ 2-point increase in FOIS scores was significantly higher in the experimental group than in the control group at both post-treatment (80.00% vs. 20.00%, *p* < 0.01) and one-month follow-up (100.00% vs. 46.67%, *p* < 0.01).

**Figure 4.**
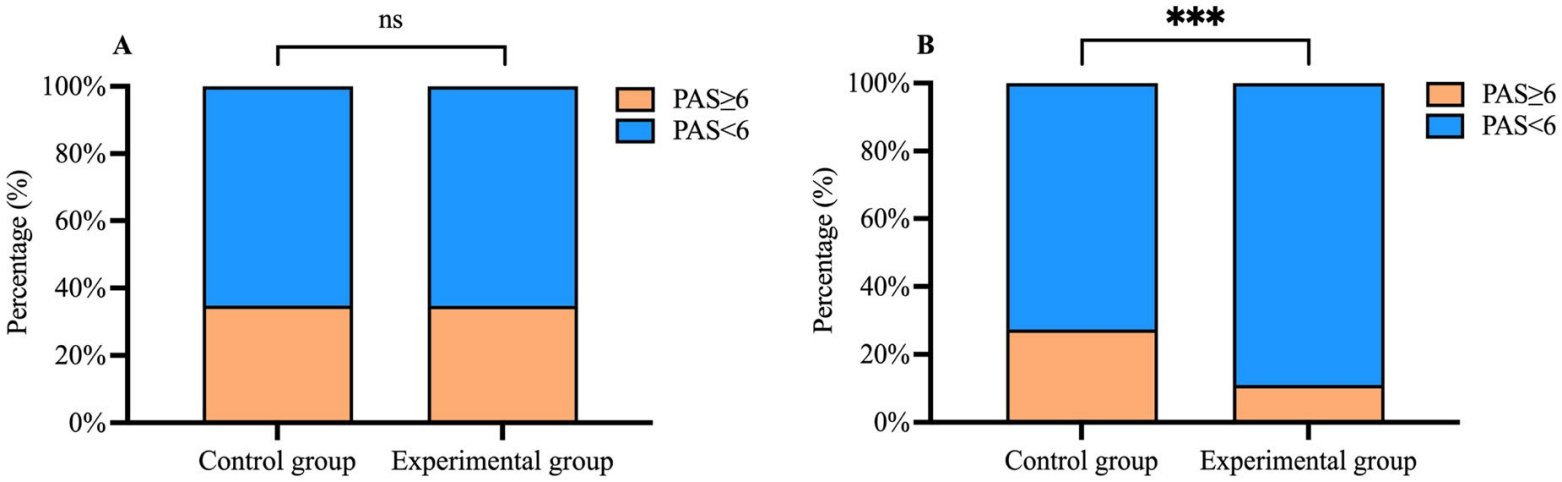
Changes in Penetration-Aspiration Scale (PAS) scores before and after intervention in both groups. (A) Percentage of PAS scores ≥6 in both groups before treatment; (B) Percentage of PAS scores ≥6 in both groups after treatment. ‘ns’ indicates that the difference is not statistically significant (*p* > 0.05), and ‘***’ indicates that the difference is statistically significant (*p* < 0.001).

Before treatment, there was no significant difference in the percentage of PAS scores ≥6 between the control and experimental groups (*p* = 0.991). At two weeks post-treatment, the percentage of PAS scores ≥6 decreased in both groups, but only the experimental group showed a statistically significant reduction (control group: 34.78% to 27.35%, *p* = 0.221; experimental group: 34.71% to 11.03%, *p* < 0.001). After treatment, the percentage of PAS scores ≥6 in the experimental group was significantly lower than in the control group (*p* < 0.001) (Figure 4).

**Figure 5.**
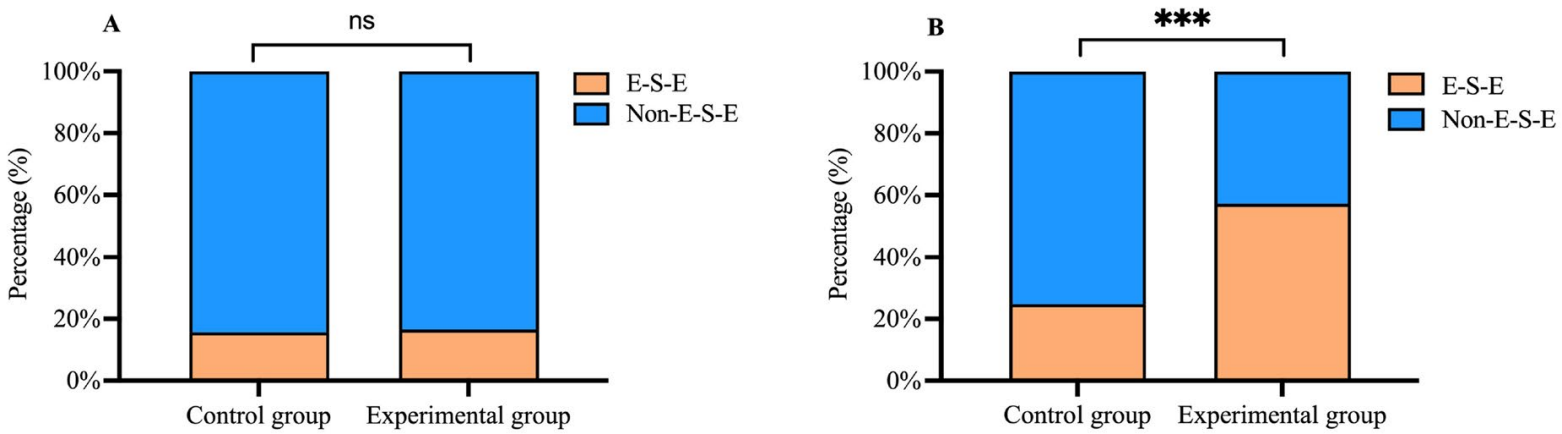
The percentage of the Exhale–Swallow–Exhale (E-SW-E) pattern before and after intervention in both groups. (A) The percentages of E-SW-E pattern in the two groups before treatment; (B) The percentages of E-SW-E pattern in the two groups after treatment. ‘ns’ indicates that the difference is not statistically significant (*p* > 0.05), and ‘***’ indicates that the difference is statistically significant (*p* < 0.001).

Before treatment, there was no significant difference in the percentage of the E-SW-E pattern between the control and experimental groups (*p* = 0.855). After treatment, the percentage of the E-SW-E pattern increased in both groups but was statistically significant only in the experimental group (control group: 15.65% to 24.79%, *p* = 0.084; experimental group: 16.53% to 57.24%, *p* < 0.001). The percentage of the E-SW-E pattern was significantly higher in the experimental group than in the control group after treatment (*p* < 0.001) (Figure 5).

Before treatment, there was no statistically significant difference in VFSS temporal and kinematic parameters between the two groups (*p* > 0.05). After treatment, in the control group, pharyngeal constriction ratio increased significantly compared to baseline (*p* = 0.012), while other parameters showed no significant changes (*p* > 0.05). In the experimental group, laryngeal vestibule closure duration (*p* = 0.035) and pharyngeal constriction ratio (*p* = 0.011) increased significantly compared to baseline, while other parameters showed no significant changes (*p* > 0.05). There were no statistically significant differences in VFSS temporal and kinematic parameters between both groups after treatment (*p* > 0.05).

## Discussion

Respiratory-swallow coordination is essential for swallowing safety but often impaired after stroke [16,17,34,35]. A significant clinical problem is the lack of rehabilitative strategies that effectively target this impairment. Accordingly, we developed and evaluated a novel bimodal biofeedback-assisted respiratory-swallow coordination training protocol for patients with post-stroke dysphagia. We found that this intervention led to a significant increase in the protective E-SW-E pattern, reduced aspiration, and enhanced functional oral intake, demonstrating superior efficacy compared to conventional therapy alone.

Swallowing in healthy adults typically occurs during expiration, likely because it provides favorable conditions for subglottic pressure formation [1,36]. According to the ‘subglottic pressure theory’ proposed by Gross et al. the mechanoreceptors on the tracheal wall can sense the subglottic pressure and transmit the sensory information *via* the afferent nerves to the central nervous system, and therefore regulate the respiratory and swallowing coordination to reduce aspiration risk [1,36,37]. However, the physiological mechanisms of swallow initiation during expiration vary across lung volume states. At high lung volumes (approaching total lung capacity), although greater subglottic pressure is generated, diaphragmatic contraction may excessively stretch the larynx, pharynx, and esophagus, potentially affecting airway protection mechanisms and swallowing dynamics [34]. At extremely low lung volumes (approaching residual volume), subglottic pressure may be insufficient [1,36]. In contrast, initiating swallowing at lung volumes slightly above functional residual capacity not only generates appropriate subglottic pressure but also facilitates key movements such as laryngeal elevation, vestibular closure, and upper esophageal sphincter opening [13,34,38].

Post-swallow expiration effectively reduces the risk of residue entering the airway. Additionally, the subglottic pressure generated during expiration helps patients perceive residue in the laryngeal vestibule and surrounding areas, triggering protective reflexes such as throat clearing or coughing [7,11,39]. The E-SW-E pattern used in this study aligns with the aforementioned research findings and physiological mechanisms, forming a crucial foundation for the therapeutic efficacy of such training.

In our study, the experimental group showed significant improvement in pharyngeal contraction rate and laryngeal vestibule closure duration compared to pre-treatment, suggesting that this training helps enhance pharyngeal contraction and improve airway protection. No significant differences were observed in other VFSS parameters between pre- and post-treatment or between the experimental and control groups. The possible explanations for this were twofold: First, VFSS temporal and kinematic parameters primarily reflect subtle changes in swallowing physiology [40–42], and the two-week intervention may not be enough to induce significant physiological remodeling. Second, the use of the 5 mL medium-viscosity bolus in the VFSS may not comprehensively reflect the intervention’s impact on swallowing physiology. Nevertheless, the improvements in FOIS score, respiratory-swallow coordination, and swallowing safety suggest that bimodal signal biofeedback-based respiratory-swallow coordination training can improve swallowing function.

Although conventional swallowing therapy plays an important role in dysphagia rehabilitation, its effectiveness is limited by the inability to provide real-time visual feedback. Previous studies have established that biofeedback, particularly surface electromyography, can effectively improve swallowing function by providing feedback on muscle activation. However, such single-modality approaches still lack the precise input needed for patients to perceive and correct respiratory-swallow patterning errors during training [43]. Building upon preliminary evidence from Martin-Harris et al. [27] in head and neck cancer patients, our study employs a randomized controlled trial design to establish more robust evidence for respiratory-swallow training in stroke-related dysphagia. In our study, we applied bimodal (respiratory-swallow) biofeedback, which overcomes the aforementioned limitation by converting physiological signals into real-time visual feedback. This method enables patients to better perceive their own respiratory-swallow pattern, recognize deviations, and make timely adjustments to accurately execute the target E-SW-E pattern. This process enhances motor learning, patient engagement, and self-awareness of swallowing action, as supported by previous studies [21,44,45].

It is important to consider that spontaneous recovery likely contributed to the functional improvements observed in both groups. Despite this, the significant between-group differences, with the experimental group demonstrating superior outcomes, indicate that the biofeedback training provided an additive therapeutic effect superimposed on the natural recovery process.

This study has several limitations. First, the small sample size and single-center design may limit the generalizability of our findings. Furthermore, the study included both ischemic and hemorrhagic stroke patients, but the limited sample size precluded stratified analyses based on stroke type or lesion location. Therefore, future multi-center studies with larger cohorts are needed not only to validate the clinical efficacy but also to explore potential variations in treatment response across different patient subgroups. Second, the follow-up period was relatively short, and long-term effects were not assessed. Future studies should extend the follow-up duration to evaluate the sustained improvement in swallowing function. Finally, this study only explored the effects of bimodal signal biofeedback-based respiratory-swallow coordination training on post-stroke dysphagia, without delving into its potential mechanisms. Future research should investigate these mechanisms to facilitate the clinical application and widespread adoption of this method.

## Conclusion

This study used a nasal airflow sensor and an accelerometer to provide bimodal signals for biofeedback, assisting patients in respiratory-swallow coordination training. The experimental group showed significant improvements in respiratory-swallow coordination and swallowing function compared to the control group. By providing real-time visual feedback, our method enables patients to intuitively perceive their respiratory-swallow patterns and actively adjust them toward the precise target pattern. Our findings support the use of bimodal signals biofeedback-based respiratory-swallow coordination training as a valuable and promising therapeutic method for post-stroke dysphagia. Future research should involve larger-scale, multi-center trials and explore the efficacy of this method across diverse patient populations to further validate and generalize its clinical application.

## Data Availability

The data support the findings of this study were shown in the paper, further inquiries can contact to the corresponding author.
